# The Effects of Polychlorinated Biphenyl Exposure During Adolescence on the Nervous System: A Comprehensive Review

**DOI:** 10.1021/acs.chemrestox.1c00226

**Published:** 2021-09-07

**Authors:** Amanda J. Bullert, Jonathan A. Doorn, Hanna E. Stevens, Hans-Joachim Lehmler

**Affiliations:** ^†^Interdisciplinary Graduate Program in Neurosciences, ^‡^Department of Occupational and Environmental Health, ^§^Department of Pharmaceutical Sciences and Experimental Therapeutics, ^∥^Department of Psychiatry, University of Iowa, Iowa City, Iowa 52242, United States

## Abstract

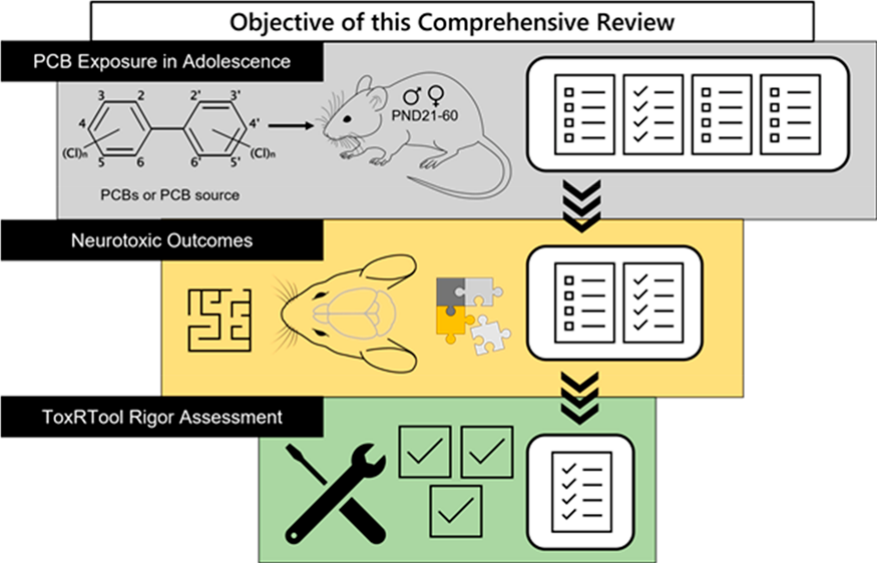

Exposure to polychlorinated biphenyls (PCBs) is implicated in adverse neurotoxic outcomes. However, the impact of PCBs on the adolescent nervous system has received inadequate attention. We conducted a comprehensive review to identify studies of neurotoxic outcomes following PCB exposure during the adolescent period in rodents. Only four papers were found to meet all inclusion criteria. PCB exposure in adolescent rats caused disruptions in the main functions of the prefrontal cortex, resulting in cognitive deficits. This comprehensive review demonstrates that more research is needed to characterize how PCB exposure adversely affects the adolescent nervous system.

## 1 Introduction

Polychlorinated biphenyls (PCBs) are a class of 209 industrial chemicals that contain a biphenyl moiety with 1–10 chlorine substituents. In the 1970s, the manufacturing of PCBs was banned in the United States. However, PCBs are still used and found in electrical equipment, building materials, and other applications, leading to their ubiquitous presence in the environment.^1,2^ PCBs can be detected in serum and adipose samples of diverse human populations. In the United States, recent studies show that PCBs are present in the indoor air of older schools due to their release from building materials.^1^ This finding is especially problematic for children who spend several hours a day in PCB-contaminated classrooms. The adolescent time frame is critical for mammalian brain development, including synaptic pruning, hormonal influences, and behavioral adaptations that underlie maturation into adulthood.^3,4^ Thus, exposure to PCBs in schools is predicted to affect the adolescent brain and prevent students from reaching their full academic potential.^2^ However, limited information is available about neurotoxic outcomes following exposure to PCBs during adolescence. Although PCB exposure is lifelong, characterizing adolescence as a window of susceptibility is important for understanding PCB neurotoxicity across the lifetime.

## 2 Identification of Studies of Neurotoxic Outcomes Following Adolescent PCB Exposure

**Figure 1:**
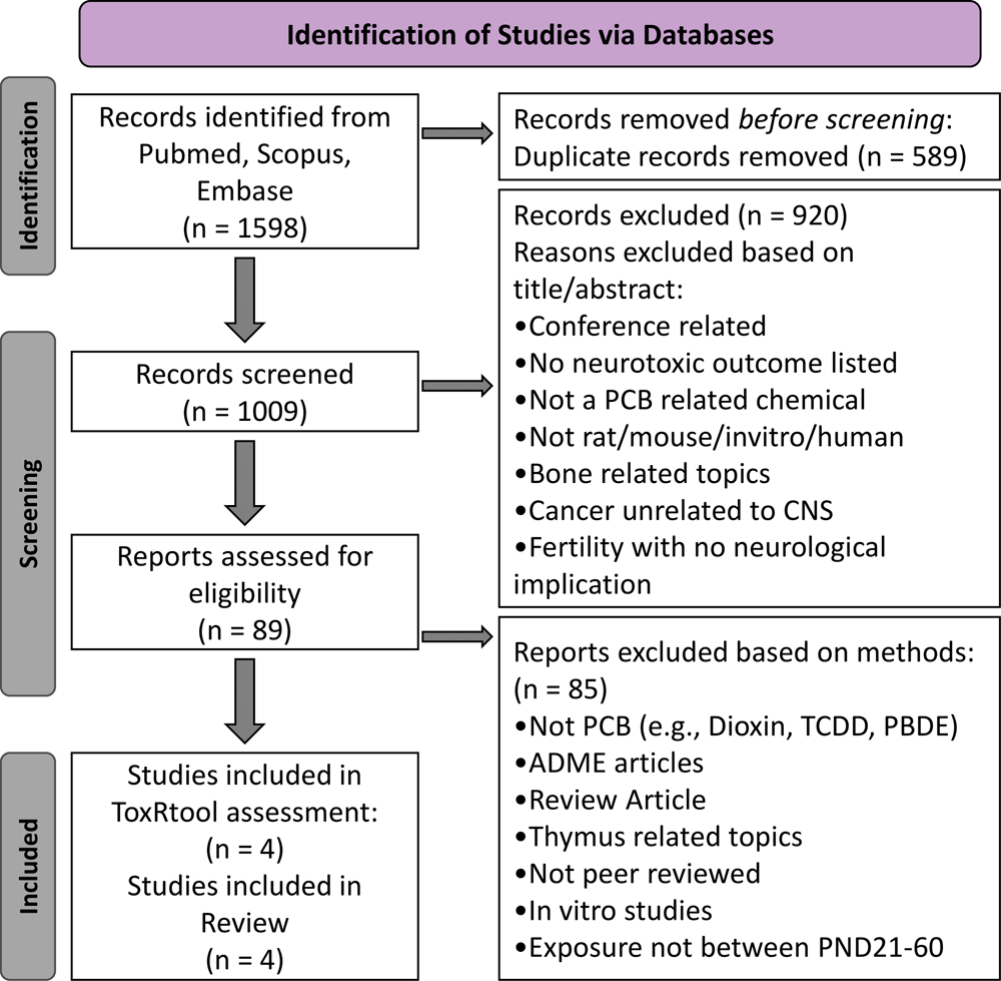
Preferred reporting items for systematic reviews and meta-analyses (PRISMA) flow diagram outlining the comprehensive review methods and criteria. The flowchart was prepared following the PRISMA 2020 statement.^15^

The objective of this comprehensive review was to identify preclinical studies that characterized neurotoxic outcomes following PCB exposure during the adolescent period of either rats or mice because of their importance in neurotoxicology research. Rodent adolescence is typically postnatal days (PND) 28–55.^4^ However, early PCB exposure can alter adolescent timelines, with male rats taking longer to reach full adult maturity and female rats developing earlier with precocious menarche.^1^ Thus, we defined adolescence in rodents as PND21–PND60, expanding the window of adolescence to encapsulate the full range of potential brain growth during this period. This age range in rodents is relevant to children in schools, from kindergarten, age 5, to high school, age 18–19.^4^ Using this broader definition of adolescence, we performed a comprehensive review and evaluated the scientific rigor of relevant papers using the ToxRtool, an open-access, user-friendly tool to evaluate toxicology studies (Figure 1).^5−8^

Pubmed, Scopus, and Embase were searched with broad Boolean terms to identify all potentially relevant studies. The search terms were generated with the help of a librarian (for additional details, see the Supporting Information). The search identified 1598 potential citations that were imported into EndNote and screened for duplicates.^9^ A total of 589 duplicates were removed. The remaining 1009 articles were evaluated based on their titles, abstracts, and methods to identify manuscripts with PCB exposure during the adolescent period for rodents and reported neurotoxic findings in adolescence or adulthood. Review criteria were predetermined to avoid bias (Figure 1). Four papers met all of the criteria. As described in the Supporting Information, scientific rigor was then evaluated with the open access ToxRtool.^5−8^ The key findings from these studies, including their ToxRtool rating, are discussed below (Table 1).

**Table 1:**
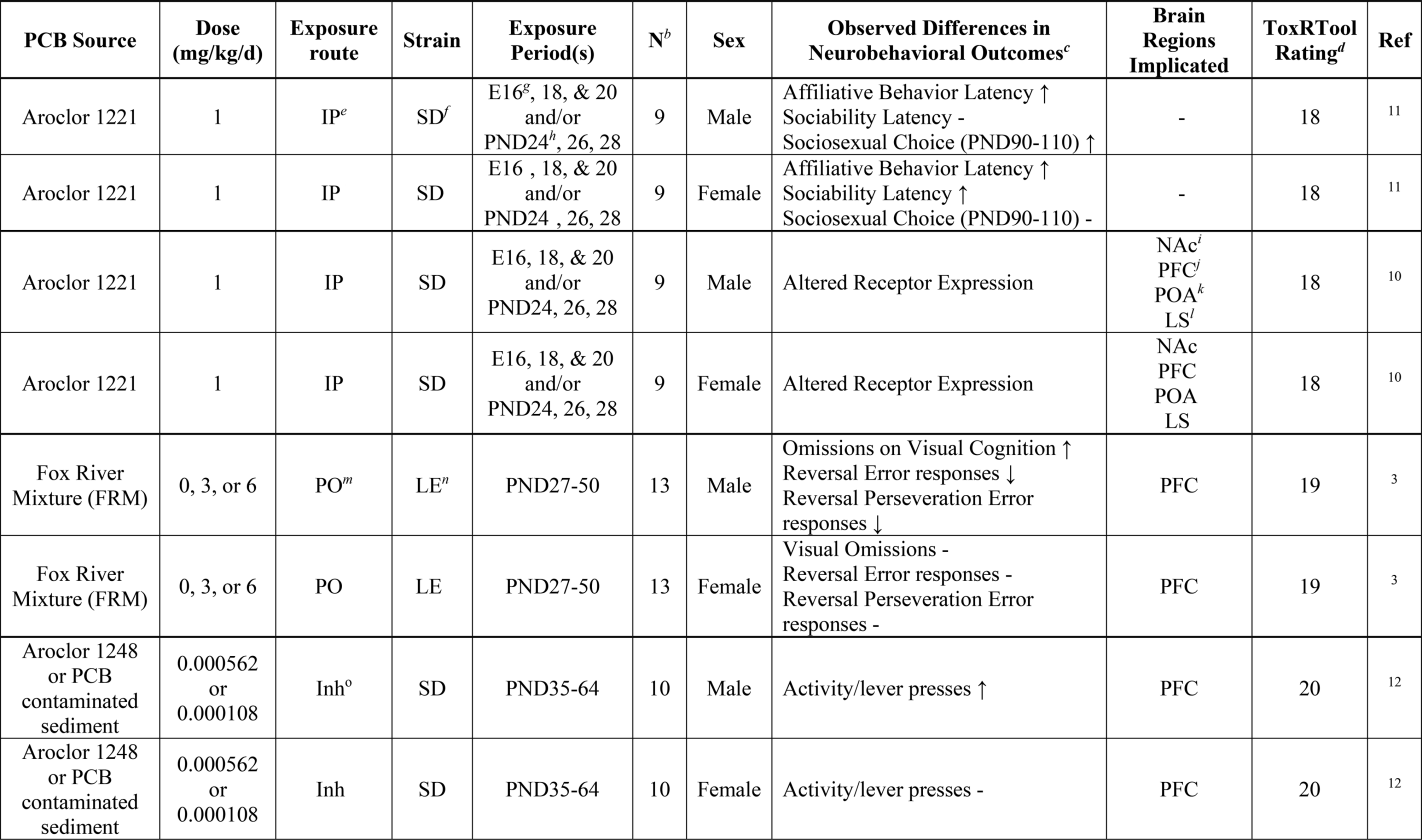
Summary of Animal Studies Investigating Neurotoxic Outcomes Following PCB Exposure during Adolescence^a^

a For additional details, see Table S2.

b *N*, number of animals per group and sex.

c (−) no change; (↑) significant increase; (↓) significant decrease.

d Tool to report quality of toxicology results^8^ (>17, reliable without restrictions; 13–17, reliable with restrictions; <13, unreliable).

e IP, Intraperitoneal injection.

f SD, Sprague Dawley.

g E, embryonic day.

h PND, postnatal day.

i NAc, nucleus accumbens.

j PFC, prefrontal cortex.

k POA, preoptic area.

l LS, lateral septum.

m PO, oral.

n LE, Long Evans.

o Inh, inhalation; whole-body inhalation.

## 3 Overview of Neurotoxic Outcomes Observed in Rodents Following PCB Exposure during Adolescence

A preclinical study investigated how exposure to PCBs first perinatally and/or later in adolescence negatively impacts behavioral and molecular outcomes in a sex and age-specific manner.^10,11^ A “two-hit” model was used to test this hypothesis. Briefly, Sprague–Dawley rats were exposed prenatally (embryonic days 16, 18, and 20) or in adolescence (PND24, PND26, and PND28) or at both time points to 1 mg/kg/bw of Aroclor^1,2^ 1221 via intraperitoneal (IP) injection. Offspring were tested behaviorally during adolescence (PND30–PND39) and adulthood (between PND90 and PND110) to assess different domains of neural function.^11^ The results listed here focus primarily on adolescent-only exposure (for a more comprehensive list of results, see Table S2). Exposure for females during adolescent development led to a longer latency to hop during affiliative behaviors along with a longer latency to socialize with a stimulus animal. In adolescent-only exposed males, no adolescent behaviors were significantly altered, but during adulthood, exposed males spent more time with females during sociosexual choice.^11^

Adolescent exposure also affected gene expression and DNA-methylation in an accompanying paper.^10^ Specifically, in lateral septum (LS), adolescent exposure altered gene expression of the androgen receptor (*Ar*) and the vasopressin receptor 1a (*Avpr1a*). Adolescent PCB exposure in males increased gene expression of the mu opioid receptor in prefrontal cortex (PFC, *Oprm1*) and decreased expression of *Avpr1a* in LS. Adolescent-only exposure also reduced expression of *Ar* and *Oprm1* in the male preoptic area (POA).^10^ The main effect from adolescent exposure in females was an increase of methylation of the *Ar* in the POA.^10^ Pearson correlations were measured to determine whether adult behaviors in males correlated with gene expression changes from adolescent PCB exposure. A significant positive correlation was reported between increased PFC *Oprm1* expression and increased time males spent near a sociosexual partner in the adolescent-only exposure group.^10,11^ Notably, the two-hit paradigm revealed complex interactions between PCB effects on the brain when exposed at multiple time points during development. While this study provides valuable insights into neurotoxic outcomes following PCB exposure during the adolescent period, the IP route of exposure is less relevant to humans, thus limiting the impact of this study.

Another study orally exposed Long Evans rats from PND27–PND50 to 0, 3, or 6 mg/kg/bw/day of the Fox River PCB mixture (FRM). This mixture mimics the PCB congener profile found in fish consumed in the Green Bay, Wisconsin region. The objective of this study was to determine whether executive functioning tasks driven by the PFC are affected in PCB exposed rats.^3^ Males exposed to the FRM displayed higher rates of cognitive flexibility (less errors on reversal learning) compared to controls in the set-shifting task, but no group differences in response inhibition.^3^ Female behavior at PND90 was not affected by adolescent exposure.

A final study investigated how PCBs affect executive functioning using operant behavior tasks.^12^ Sprague–Dawley rats inhaled an estimated 0.562 mg/kg/day of Aroclor 1248 vapor, a commercial PCB mixture, or vapors from PCB-contaminated sediment from the St. Lawrence River from PND35–PND65. These exposure paradigms are representative of current human exposures to PCBs in schools. Inhalation exposure to either Aroclor 1248 or PCB contaminated sediment affected both male and female performance during fixed interval trials. The exposed males expressed reduced inhibition and lower control of responses (more activity and lever pressing) compared to controls. The females, although not statistically significant, responded less frequently in general than the control littermates during fixed interval trials.^12^

The available evidence, while limited, demonstrates that the PFC and behavioral tasks that rely on the PFC are influenced following exposure to PCBs during adolescence.^1,3,10−12^ The PFC is one of the major areas undergoing change and development during adolescence. The PFC is responsible for complex human behaviors, including socializing, critical thinking, decision making, and regulating reward responses.^3^ In the preclinical studies identified in this review, PCB exposure during adolescence caused disruptions in critical functions of the PFC as evidenced by increased latency to socializing in females,^11^ increased activity and impulsivity in males during exploration,^11,12^ and males showing changes in set-shifting abilities (Table S2).^3^ These changes are associated with altered gene expression of *Oprm1* in the PFC.

The higher-order functions displayed by the PFC and its associated circuits require proper adolescent developmental processes, which can be disrupted following PCB exposure. Adolescent PFC development is heavily influenced by a late wave dopaminergic innervation which plays a critical role in the social, motor, and cognitive behaviors found to be disrupted by PCBs in these studies. Disruption of dopamine signaling can lead to behavioral dysregulation like impulsivity, addiction, and maladaptive habit formation.^12^ PCB exposure can affect dopamine levels, especially in adult mammals.^13,14^ Thus, disruption in dopamine circuits in the PFC following PCB exposure during adolescence may be a factor in adverse behavioral outcomes during adolescence and later in adulthood observed in the preclinical studies identified by this comprehensive review;^12^ however, changes in dopamine levels following PCB exposure during adolescence were not assessed in the studies discussed in this comprehensive review.

## 4 Knowledge Gaps and Research Needs

Because adolescents continue to be exposed to PCBs via their diet and by inhalation,^1^ there is a need to characterize further sex- and dose-dependent effects of PCB exposure on the adolescent brain using relevant routes of exposure and environmentally relevant doses. For example, it is unknown how PCBs and their metabolites accumulate in different brain regions; the mechanisms (e.g., altered neurotransmitter homeostasis) by which PCBs affect cellular targets in the adolescent brain have not been characterized; and the circuits involved in adverse behavioral outcomes following PCB exposure have not been studied. Moreover, behavioral studies assessing cognitive deficits through behavioral outcomes in preclinical animal models are needed to translate laboratory findings to humans. Without answering these knowledge gaps, it will be impossible to prevent and mitigate the adverse neurotoxic effects of PCBs in future generations.

## Supporting Information Available

Description of the comprehensive review, database search terms, and summary of biological end points investigated in studies identified in this comprehensive review (PDF)

## Abbreviations

Ar: Androgen receptor
E: embryonic
Inh: inhalation
IP: intraperitoneal injection
LE: Long Evans
LS: lateral septum
NAc: nucleus accumbens
PCB: polychlorinated biphenyl
PFC: prefrontal cortex
PND: postnatal day
PO: oral
POA: preoptic area
SD: Sprague–Dawley
