# Supplementary material for: The Effects of Polychlorinated Biphenyl Exposure During Adolescence on the Nervous System: A Comprehensive Review
Source: Chem Res Toxicol. 2021 Sep 7;34(9):1948–52. doi: 10.1021/acs.chemrestox.1c00226 (PMC8456429; doi:10.1021/acs.chemrestox.1c00226)
Supplement: Supplementary file 1 — tx1c00226_si_001.pdf [file tx1c00226_si_001.pdf]

SUPPORTING INFORMATION

The Effects of Polychlorinated Biphenyl Exposure During Adolescence on the Nervous System: A Comprehensive Review

Amanda J. Bullert[a,b], Jonathan A. Doorn[a,c], Hanna Stevens[a,d], and Hans-Joachim Lehmler[a,b] *

[a]Interdisciplinary Graduate Program in Neurosciences, [b]Department of Occupational and Environmental Health, [c]Department of Pharmaceutical Sciences and Experimental Therapeutics, [d]Department of Psychiatry, University of Iowa, Iowa City, Iowa 52242, USA

*Corresponding Author:
Dr. Hans-Joachim Lehmler
The University of Iowa
Department of Occupational and Environmental Health
University of Iowa Research Park, #164 MTF
Iowa City, IA 52242-5000
Phone: (319) 335-4981
Fax: (319) 335-4290
e-mail: hans-joachim-lehmler@uiowa.edu

Table of Contents

Methods and scoping search S2

Table S1. Search terms and categories S4

Table S2. Extended summary of all neurotoxic outcomes listed in the four papers included in the comprehensive review S8

References S10

Methods for the comprehensive review: First, we developed an *a priori* protocol for all stages of the comprehensive review, including selecting terms, conducting the review process, determining inclusion and exclusion criteria, filtering studies, and reviewing results. At least two reviewers participated in each phase of the comprehensive review process[1].

Term selection: Boolean terms were used to search the Pubmed, Scopus, and Embase databases for relevant citations. The terms used in the comprehensive search were determined based on all possible variations of the terms using vectors corresponding with the respective databases: Pubmed: MeSh, Embase: Emtree, and Scopus: NA. A librarian verified the search strategies to ensure all possible search terms to determine potential gaps in preclinical studies with adolescent PCB exposure and neurotoxic outcomes. All terms were input into each database to reduce bias. The librarian verified that the Boolean terms and database vectors were used appropriately[2]. Terms were then organized in Table S1 below to align the corresponding terms to the search category. The search strategies for each database can be found below.

Additional information about the comprehensive review methods. The inclusion and exclusion criteria for evaluating all publications identified through the database searches were selected for two different phases. Phase one was designed to have criteria that eliminated citations that were clearly not relevant to the objective of the comprehensive review. Electronic copies of the manuscripts identified for inclusion in phase one were obtained and screened broadly in phase two for adverse effects of PCBs on the brain or central nervous system following *in vivo* exposure during the adolescent period as defined for the purpose of this comprehensive review. Murine species were selected for inclusion in the comprehensive review because the goal was to focus on preclinic studies and not later translational studies. The Preferred Reporting Items for Systematic reviews and Meta-Analyses (PRISMA) flowchart was used to organize and visualize the entire review process[3].

ToxRtool: The scientific rigor of all studies identified through the review process was assessed using the ToxRTool. The ToxRtool is open access and user-friendly tool to assess reliability in scientific results[4, 5]. This tool was developed to evaluate studies reporting toxicology-based research. Criteria and ratings of the

ToxRTool are based on the Klimisch scoring system, which assigns scores from 1-4. The ToxRtool expands these criteria and categories for a more holistic look at studies reporting toxicology-based results. Scores are based on the following criteria groups: 1) Test substance identification, 2) test organism characterization, 3) study design description, 4) study results documentation, 5) plausibility of study design and results.

Table S1. Search terms and categories.

Topics	Pubmed	Embase	Scopus
Rodents	"Rats"[Mesh]<br>"Murinae"[Mesh]<br>"Mice"[Mesh]	'rat'/exp<br>'murine'/exp<br>'mouse'/exp<br>'Mus musculus'/exp<br>'Rattus rattus'/exp<br>'rodent'/exp<br>'pup (rodent)'/exp	Rodent<br>Rodents<br>Pup<br>Pups<br>“pup (rodent)”<br>Rat<br>Rats<br>Murinae<br>Rattus<br>Mus<br>“mus musculus”<br>Mouse<br>Mice<br>“rattus rattus”<br>murine
	Rodent[Title/Abstract]<br>Rodents[Title/Abstract]<br>Pup[Title/Abstract]<br>Pups[Title/Abstract]<br>“pup (rodent)” [Title/Abstract]<br>Rat[Title/Abstract]<br>Rats[Title/Abstract]<br>Murinae[Title/Abstract]<br>Rattus[Title/Abstract]<br>Mus[Title/Abstract]<br>“mus musculus” [Title/Abstract]<br>Mouse[Title/Abstract]<br>Mice[Title/Abstract]<br>“rattus rattus” [Title/Abstract]<br>murine[Title/Abstract]	Rodent:ti,ab,kw<br>rodents:ti,ab,kw<br>Pup:ti,ab,kw<br>Pups:ti,ab,kw<br>“pup (rodent)”:ti,ab,kw<br>Rat:ti,ab,kw<br>Rats:ti,ab,kw<br>Murinae:ti,ab,kw<br>Rattus:ti,ab,kw<br>Mus:ti,ab,kw<br>“mus musculus”:ti,ab,kw<br>Mouse:ti,ab,kw<br>Mice:ti,ab,kw<br>“rattus rattus”:ti,ab,kw<br>murine:ti,ab,kw	
Adolescence	"Adolescent"[Mesh]<br>"Child"[Mesh]<br>"Minors"[Mesh]	'adolescent'/exp<br>'juvenile'/exp<br>'child'/exp	Adolescen*<br>Teen*<br>Teen<br>Minor<br>Minors<br>Juvenile<br>Juveniles<br>Child*<br>child
	Adolescen*[Title/Abstract]<br>Teen*[Title/Abstract]<br>Teen[Title/Abstract]<br>Minor[Title/Abstract]<br>Minors[Title/Abstract]<br>Juvenile[Title/Abstract]<br>Juveniles[Title/Abstract]<br>Child*[Title/Abstract]<br>child[Title/Abstract]	Adolescen*:ti,ab,kw<br>Teen*:ti,ab,kw<br>Teen:ti,ab,kw<br>Minor:ti,ab,kw<br>Minors:ti,ab,kw<br>Juvenile:ti,ab,kw<br>Juveniles:ti,ab,kw<br>Child*:ti,ab,kw<br>child:ti,ab,kw	
PCB	"Polychlorinated Biphenyls"[Mesh]<br>"Biphenyl Compounds"[Mesh]<br>"Aroclors"[Mesh]<br>"Dioxins and Dioxin-like Compounds"[Mesh]<br>"Dioxins"[Mesh]	'polychlorinated biphenyl'/exp<br>'biphenyl derivative'/exp<br>'aroclor'/exp<br>'dioxin'/exp	PCB<br>PCBs<br>“Polychlorinated Biphenyls”<br>“Polychlorinated Biphenyl”<br>“Biphenyl compound”<br>“biphenyl compounds”<br>Aroclor<br>Aroclors<br>"Dioxins and Dioxin-like Compounds"<br>Dioxin<br>Dioxins<br>“biphenyl derivative”
	PCB[Title/Abstract]<br>PCBs[Title/Abstract]<br>“Polychlorinated Biphenyls” [Title/Abstract]<br>“Polychlorinated Biphenyl” [Title/Abstract]<br>“Biphenyl compound” [Title/Abstract]<br>“biphenyl compounds” [Title/Abstract]<br>Aroclor[Title/Abstract]<br>Aroclors[Title/Abstract]<br>"Dioxins and Dioxin-like Compounds"[Title/Abstract]<br>Dioxin[Title/Abstract]<br>Dioxins[Title/Abstract]<br>“biphenyl derivative” [Title/Abstract]	PCB:ti,ab,kw<br>PCBs:ti,ab,kw<br>“Polychlorinated Biphenyls”:ti,ab,kw<br>“Polychlorinated Biphenyl”:ti,ab,kw<br>“Biphenyl compound”:ti,ab,kw<br>“biphenyl compounds”:ti,ab,kw<br>Aroclor:ti,ab,kw<br>Aroclors:ti,ab,kw<br>"Dioxins and Dioxin-like Compounds":ti,ab,kw<br>Dioxin:ti,ab,kw<br>Dioxins:ti,ab,kw<br>“biphenyl derivative”:ti,ab,kw	

Table S1 – continued. Search terms and categories.

<table>
<tr><th>Topics</th><th>Pubmed</th><th>Embase</th><th>Scopus</th></tr>
<tr><td rowspan="2">Neuro</td><td>"Attention Deficit Disorder with Hyperactivity"[Mesh]
"Neurodevelopmental Disorders"[Mesh]
"toxicity" [Subheading]
"Neurocognitive Disorders"[Mesh]
"Growth and Development"[Mesh]
"Brain"[Mesh]
"Central Nervous System"[Mesh]
"Nervous System"[Mesh]</td><td>'attention deficit disorder'/exp
'mental disease'/exp
'toxicity'/exp
'growth, development and aging'/exp
'brain'/exp
'central nervous system'/exp
'nervous system'/exp</td><td rowspan="2">"Attention Deficit Disorder with Hyperactivity"
“Attention Deficit Disorder”
ADHD
Toxic
Toxi*
“neurodevelopment disorders”
“neurodevelopment disorder”
Neurotox*
Neurodeve*
"Neurocognitive Disorders"
"Neurocognitive Disorder"
“Mental disease”
Neurocogn*
"Growth and Development"
Growth
Develop*
Develop
Brain
Brains
"Central Nervous System"
CNS
"Nervous System"
“growth, development and aging”
“attention deficit hyperactivity disorder”</td></tr>
<tr><td>"Attention Deficit Disorder with Hyperactivity"[Title/Abstract]
“Attention Deficit Disorder” [Title/Abstract]
ADHD[Title/Abstract]
Toxic[Title/Abstract]
Toxi*[Title/Abstract]
“neurodevelopment disorders” [Title/Abstract]
“neurodevelopment disorder” [Title/Abstract]
Neurotox*[Title/Abstract]
Neurodeve*[Title/Abstract]
"Neurocognitive Disorders"[Title/Abstract]
"Neurocognitive Disorder"[Title/Abstract]
“Mental disease” [Title/Abstract]
Neurocogn*[Title/Abstract]
"Growth and Development"[Title/Abstract]
Growth[Title/Abstract]
Develop*[Title/Abstract]
Develop[Title/Abstract]
Brain[Title/Abstract]
Brains[Title/Abstract]
"Central Nervous System"[Title/Abstract]
CNS[Title/Abstract]
"Nervous System"[Title/Abstract]
“growth, development and aging” [Title/Abstract]
“attention deficit hyperactivity disorder”[Title/Abstract]</td><td>"Attention Deficit Disorder with Hyperactivity":ti,ab,kw
“Attention Deficit Disorder”:ti,ab,kw
ADHD:ti,ab,kw
Toxic:ti,ab,kw
Toxi*:ti,ab,kw
“neurodevelopment disorders”:ti,ab,kw
“neurodevelopment disorder”:ti,ab,kw
Neurotox*:ti,ab,kw
Neurodeve*:ti,ab,kw
"Neurocognitive Disorders":ti,ab,kw
"Neurocognitive Disorder":ti,ab,kw
“Mental disease”:ti,ab,kw
Neurocogn*:ti,ab,kw
"Growth and Development":ti,ab,kw
Growth:ti,ab,kw
Develop*:ti,ab,kw
Develop:ti,ab,kw
Brain:ti,ab,kw
Brains:ti,ab,kw
"Central Nervous System":ti,ab,kw
CNS:ti,ab,kw
"Nervous System":ti,ab,kw
“growth, development and aging”:ti,ab,kw
“attention deficit hyperactivity disorder”:ti,ab,kw</td></tr>
</table>

Initial Pubmed Search strategy
N=341
Full search:
("Rats"[Mesh] OR "Murinae"[Mesh] OR "Mice"[Mesh] OR Rodent[Title/Abstract] OR Rodents[Title/Abstract] OR Pup[Title/Abstract] OR Pups[Title/Abstract] OR “pup (rodent)”[Title/Abstract] OR Rat[Title/Abstract] OR Rats[Title/Abstract] OR Murinae[Title/Abstract] OR Rattus[Title/Abstract] OR Mus[Title/Abstract] OR “mus musculus”[Title/Abstract] OR Mouse[Title/Abstract] OR Mice[Title/Abstract] OR “rattus rattus”[Title/Abstract] OR murine[Title/Abstract]) AND ("Adolescent"[Mesh] OR "Child"[Mesh] OR "Minors"[Mesh] OR Adolescen*[Title/Abstract] OR Teen*[Title/Abstract] OR Teen[Title/Abstract] OR Minor[Title/Abstract] OR Minors[Title/Abstract] OR Juvenile[Title/Abstract] OR Juveniles[Title/Abstract] OR Child*[Title/Abstract] OR child[Title/Abstract]) AND ("Polychlorinated Biphenyls"[Mesh] OR "Biphenyl Compounds"[Mesh] OR "Aroclors"[Mesh] OR "Dioxins and Dioxin-like Compounds"[Mesh] OR "Dioxins"[Mesh] OR PCB[Title/Abstract] OR PCBs[Title/Abstract] OR “Polychlorinated Biphenyls”[Title/Abstract] OR “Polychlorinated Biphenyl”[Title/Abstract] OR “Biphenyl compound”[Title/Abstract] OR “biphenyl compounds”[Title/Abstract] OR Aroclor[Title/Abstract] OR Aroclors[Title/Abstract] OR "Dioxins and Dioxin-like Compounds"[Title/Abstract] OR Dioxin[Title/Abstract] OR Dioxins[Title/Abstract] OR “biphenyl derivative”[Title/Abstract]) AND ("Attention Deficit Disorder with Hyperactivity"[Mesh] OR "Neurodevelopmental Disorders"[Mesh] OR "toxicity"[Subheading] OR "Neurocognitive Disorders"[Mesh] OR "Growth and Development"[Mesh] OR "Brain"[Mesh] OR "Central Nervous System"[Mesh] OR "Nervous System"[Mesh] OR "Attention Deficit Disorder with Hyperactivity"[Title/Abstract] OR “attention deficit hyperactivity disorder”[Title/Abstract] OR “Attention Deficit Disorder”[Title/Abstract] OR ADHD[Title/Abstract] OR Toxic[Title/Abstract] OR Toxi*[Title/Abstract] OR “neurodevelopment disorders”[Title/Abstract] OR “neurodevelopment disorder”[Title/Abstract] OR Neurotox*[Title/Abstract] OR Neurodeve*[Title/Abstract] OR "Neurocognitive Disorders"[Title/Abstract] OR "Neurocognitive Disorder"[Title/Abstract] OR “Mental disease”[Title/Abstract] OR Neurocogn*[Title/Abstract] OR "Growth and Development"[Title/Abstract] OR Growth[Title/Abstract] OR Develop*[Title/Abstract] OR Develop[Title/Abstract] OR Brain[Title/Abstract] OR Brains[Title/Abstract] OR "Central Nervous System"[Title/Abstract] OR CNS[Title/Abstract] OR "Nervous System"[Title/Abstract] OR “growth, development and aging”[Title/Abstract])

Initial EMBASE Search strategy
N=843
Full search:
('rat'/exp OR 'murine'/exp OR 'mouse'/exp OR 'Mus musculus'/exp OR 'Rattus rattus'/exp OR 'rodent'/exp OR 'pup (rodent)'/exp OR Rodent:ti,ab,kw OR rodents:ti,ab,kw OR Pup:ti,ab,kw OR Pups:ti,ab,kw OR “pup (rodent)”:ti,ab,kw OR Rat:ti,ab,kw OR Rats:ti,ab,kw OR Murinae:ti,ab,kw OR Rattus:ti,ab,kw OR Mus:ti,ab,kw OR “mus musculus”:ti,ab,kw OR Mouse:ti,ab,kw OR Mice:ti,ab,kw OR “rattus rattus”:ti,ab,kw OR murine:ti,ab,kw) AND ('adolescent'/exp OR 'juvenile'/exp OR 'child'/exp OR Adolescen*:ti,ab,kw OR Teen*:ti,ab,kw OR Teen:ti,ab,kw OR Minor:ti,ab,kw OR Minors:ti,ab,kw OR Juvenile:ti,ab,kw OR Juveniles:ti,ab,kw OR Child*:ti,ab,kw OR child:ti,ab,kw) AND ('polychlorinated biphenyl'/exp OR 'biphenyl derivative'/exp OR 'aroclor'/exp OR 'dioxin'/exp OR PCB:ti,ab,kw OR PCBs:ti,ab,kw OR “Polychlorinated Biphenyls”:ti,ab,kw OR “Polychlorinated Biphenyl”:ti,ab,kw OR “Biphenyl compound”:ti,ab,kw OR “biphenyl compounds”:ti,ab,kw OR Aroclor:ti,ab,kw OR Aroclors:ti,ab,kw OR "Dioxins and Dioxin-like Compounds":ti,ab,kw OR Dioxin:ti,ab,kw OR Dioxins:ti,ab,kw OR “biphenyl derivative”:ti,ab,kw) AND ('attention deficit disorder'/exp OR 'mental disease'/exp OR 'toxicity'/exp OR 'growth, development and aging'/exp OR 'brain'/exp OR 'central nervous system'/exp OR 'nervous system'/exp OR “Attention Deficit Disorder with Hyperactivity":ti,ab,kw OR “attention deficit hyperactivity disorder”:ti,ab,kw OR “Attention Deficit Disorder”:ti,ab,kw OR ADHD:ti,ab,kw OR Toxic:ti,ab,kw OR Toxi*:ti,ab,kw OR “neurodevelopment disorders”:ti,ab,kw OR “neurodevelopment disorder”:ti,ab,kw OR Neurotox*:ti,ab,kw OR Neurodeve*:ti,ab,kw OR "Neurocognitive Disorders":ti,ab,kw OR "Neurocognitive Disorder":ti,ab,kw OR “Mental disease”:ti,ab,kw OR Neurocogn*:ti,ab,kw OR "Growth and Development":ti,ab,kw OR Growth:ti,ab,kw OR Develop*:ti,ab,kw OR Develop:ti,ab,kw OR Brain:ti,ab,kw OR Brains:ti,ab,kw OR "Central Nervous System":ti,ab,kw OR CNS:ti,ab,kw OR "Nervous System":ti,ab,kw OR “growth, development and aging”:ti,ab,kw)

Initial SCOPUS Search strategy
N=400
Full search:

((TITLE-ABS-KEY (rodent) OR TITLE-ABS-KEY (rodents) OR TITLE-ABS-KEY (pup) OR TITLE-ABS-KEY (pups) OR TITLE-ABS-KEY (rat) OR TITLE-ABS-KEY (rats) OR TITLE-ABS-KEY (murinae) OR TITLE-ABS-KEY (rattus) OR TITLE-ABS-KEY (mus) OR TITLE-ABS-KEY ("mus musculus") OR TITLE-ABS-KEY (mouse) OR TITLE-ABS-KEY (mice) OR TITLE-ABS-KEY ("rattus rattus") OR TITLE-ABS-KEY (murine))) AND ((TITLE-ABS-KEY (adolescen*) OR TITLE-ABS-KEY (teen*) OR TITLE-ABS-KEY (teen) OR TITLE-ABS-KEY (minor) OR TITLE-ABS-KEY (minors) OR TITLE-ABS-KEY (juvenile) OR TITLE-ABS-KEY (juveniles) OR TITLE-ABS-KEY (child*) OR TITLE-ABS-KEY (child))) AND ((TITLE-ABS-KEY (pcb) OR TITLE-ABS-KEY (pcbs) OR TITLE-ABS-KEY ("polychlorinated biphenyls") OR TITLE-ABS-KEY ("polychlorinated biphenyl") OR TITLE-ABS-KEY ("biphenyl compound") OR TITLE-ABS-KEY ("biphenyl compounds") OR TITLE-ABS-KEY (aroclor) OR TITLE-ABS-KEY (aroclors) OR TITLE-ABS-KEY ("Dioxins and Dioxin-like Compounds") OR TITLE-ABS-KEY (dioxin) OR TITLE-ABS-KEY (dioxins) OR TITLE-ABS-KEY ("biphenyl derivative"))) AND ((TITLE-ABS-KEY ("Attention Deficit Disorder with Hyperactivity") OR TITLE-ABS-KEY ("Attention Deficit Disorder") OR TITLE-ABS-KEY (“attention deficit hyperactivity disorder”) OR TITLE-ABS-KEY (adhd) OR TITLE-ABS-KEY (toxic) OR TITLE-ABS-KEY (toxi*) OR TITLE-ABS-KEY ("neurodevelopment disorders") OR TITLE-ABS-KEY ("neurodevelopment disorder") OR TITLE-ABS-KEY (neurotox*) OR TITLE-ABS-KEY (neurodeve*) OR TITLE-ABS-KEY ("neurocognitive disorders") OR TITLE-ABS-KEY ("neurocognitive disorder") OR TITLE-ABS-KEY ("mental disease") OR TITLE-ABS-KEY (neurocogn*) OR TITLE-ABS-KEY ("growth and development") OR TITLE-ABS-KEY (growth) OR TITLE-ABS-KEY (develop*) OR TITLE-ABS-KEY (brain) OR TITLE-ABS-KEY (brains) OR TITLE-ABS-KEY (develop) OR TITLE-ABS-KEY ("central nervous system") OR TITLE-ABS-KEY (cns) OR TITLE-ABS-KEY ("nervous system") OR TITLE-ABS-KEY ("growth, development and aging"))) AND (LIMIT-TO (LANGUAGE , "English"))

Table S2. Extended summary of all neurotoxic outcomes listed in the four papers included in the comprehensive review.

PCB Source	Exposure Group	Observed Neurological Outcomes		Reference
		**Male**	**Female**	
Aroclor 1221	Aroclor 1221 - Vehicle	Affiliative Behavior -<br>Affiliative Behavior Latency -<br>Sociability Latency -<br>Anxiety-like Behavior -<br>Sociosexual Choice ↓<br>Anxiety-like Behavior (Adult) -	Affiliative Behavior -<br>Affiliative Behavior Latency -<br>Sociability Latency -<br>Anxiety-like Behavior -<br>Sociosexual Choice -<br>Anxiety-like Behavior (Adult) -	6
	Vehicle - Aroclor 1221	Affiliative Behavior -<br>Affiliative Behavior Latency -<br>Sociability Latency -<br>Anxiety-like Behavior -<br>Sociosexual Choice ↑<br>Anxiety-like Behavior (Adult) -	Affiliative Behavior -<br>Affiliative Behavior Latency ↑<br>Sociability Latency ↑<br>Anxiety-like Behavior -<br>Sociosexual Choice -<br>Anxiety-like Behavior (Adult) -	
	Aroclor 1221 - Aroclor 1221	Affiliative Behavior -<br>Affiliative Behavior Latency ↑<br>Sociability Latency -<br>Anxiety-like Behavior -<br>Sociosexual Choice ↓<br>Anxiety-like Behavior (Adult) -	Affiliative Behavior ↑<br>Affiliative Behavior Latency ↑<br>Sociability Latency ↑<br>Anxiety-like Behavior ↓<br>Sociosexual Choice -<br>Anxiety-like Behavior (Adult) -	
Aroclor 1221	Aroclor 1221 - Vehicle	Circulating Hormone Levels: E_2 -, P_4 -, T -, T3 -, T4 -<br>DNA methylation (*Oprm1)* -<br>DNA methylation (*Ar)* -<br><u>Receptor Expression</u><br>POA: *(Ar)* ↓, *(Esr1)* -, *(Oprm1)* ↓, *(Otxr)* ↓, *(Drd2)* -, *(Avpr1a)* -<br>PFC: *(Ar)* -, *(Esr1)* -, *(Oprm1)* -, *(Otxr)* -, *(Drd2)* -, *(Avpr1a)* -<br>NAc: *(Ar)* -, *(Esr1)* -, *(Oprm1)* -, *(Otxr)* -, *(Drd2)* ↑, *(Avpr1a)* -<br>LS: *(Ar)* ↓, *(Esr1)* -, *(Oprm1)* -, *(Otxr)* -, *(Drd2)* -, *(Avpr1a)* ↓	Circulating Hormone Levels: E_2 -, P_4 -, T3 -, T4 -<br>DNA methylation (*Oprm1)* -<br>DNA methylation (*Ar)* -<br><u>Receptor Expression</u><br>POA: *(Ar)* -, *(Esr1)* -, *(Oprm1)* -, *(Otxr)* -, *(Drd2)* -, *(Avpr1a)* -<br>PFC: *(Ar)* -, *(Esr1)* -, *(Oprm1)* -, *(Otxr)* -, *(Drd2)* -, *(Avpr1a)* -<br>NAc: *(Ar)* -, *(Esr1)* -, *(Oprm1)* -, *(Otxr)* -, *(Drd2)* -, *(Avpr1a)* -<br>LS: *(Ar)* -, *(Esr1)* -, *(Oprm1)* -, *(Otxr)* -, *(Drd2)* -, *(Avpr1a)* -	7
	Vehicle - Aroclor 1221	Circulating Hormone Levels: E_2 -, P_4 -, T -, T3 -, T4 -<br>DNA methylation (*Oprm1)* -<br>DNA methylation (*Ar)* -<br><u>Receptor Expression</u><br>POA: *(Ar)* ↓, *(Esr1)* -, *(Oprm1)* ↓, *(Otxr)* -, *(Drd2)* -, *(Avpr1a)* -<br>PFC: *(Ar)* -, *(Esr1)* -, *(Oprm1)* ↑, *(Otxr)* -, *(Drd2)* -, *(Avpr1a)* -<br>NAc: *(Ar)* -, *(Esr1)* -, *(Oprm1)* -, *(Otxr)* -, *(Drd2)* -, *(Avpr1a)* -<br>LS: *(Ar)* ↓, *(Esr1)* -, *(Oprm1)* -, *(Otxr)* -, *(Drd2)* -, *(Avpr1a)* ↓	Circulating Hormone Levels: E_2 -, P_4 -, T3 -, T4 -<br>DNA methylation (*Oprm1)* -<br>DNA methylation (*Ar)* ↑<br><u>Receptor Expression</u><br>POA: *(Ar)* -, *(Esr1)* -, *(Oprm1)* -, *(Otxr)* -, *(Drd2)* -, *(Avpr1a)* -<br>PFC: *(Ar)* -, *(Esr1)* -, *(Oprm1)* -, *(Otxr)* -, *(Drd2)* -, *(Avpr1a)* -<br>NAc: *(Ar)* -, *(Esr1)* -, *(Oprm1)* -, *(Otxr)* -, *(Drd2)* -, *(Avpr1a)* -<br>LS: *(Ar)* -, *(Esr1)* -, *(Oprm1)* -, *(Otxr)* -, *(Drd2)* -, *(Avpr1a)* -	

(-) No change; (↑) Significant Increase; (↓) Significant Decrease
PFC, Prefrontal Cortex; NAc, Nucleus Accumbens; MPOA, Medial Preoptic Area; LS, Lateral Septum; Ar, Androgen Receptor; Esr1, estrogen receptor alpha; Oprm1, mu opioid receptor; Oxtr, oxytocin receptor; Drd2, dopamine receptor D2; Avpr1a, vasopressin receptor 1a; E_2, Estradiol; P_4, Progesterone; T, Testosterone; T3, Triiodothyronine; T4, Thyroxine

Table S2 – continued. Extended summary of all neurotoxic outcomes listed in the four papers included in the comprehensive review.

PCB Source	*Exposure Group*	*Observed Neurological Outcomes*		*Reference*
		**Male**	**Female**	
Aroclor 1221	Aroclor 1221 -<br>Aroclor 1221	*Circulating Hormone Levels: E_2 -, P_4 ↓, T -, T3 -, T4 -*<br>*DNA methylation (Oprm1) -*<br>*DNA methylation (Ar) -*<br>*<u>Receptor Expression</u>*<br>*POA: (Ar) -, (Esr1) ↑, (Oprm1) ↑, (Otxr) -, (Drd2) -, (Avpr1a) -*<br>*PFC: (Ar) -, (Esr1) -, (Oprm1) ↑, (Otxr) -, (Drd2) -, (Avpr1a) -*<br>*NAc: (Ar) -, (Esr1) -, (Oprm1) -, (Otxr) -, (Drd2) ↑, (Avpr1a) -*<br>*LS: (Ar) -, (Esr1) -, (Oprm1) -, (Otxr) -, (Drd2) -, (Avpr1a) -*	*Circulating Hormone Levels: E_2 -, P_4 -, T3 -, T4 -*<br>*DNA methylation (Oprm1) -*<br>*DNA methylation (Ar) ↑*<br>*<u>Receptor Expression</u>*<br>*POA: (Ar) -, (Esr1) -, (Oprm1) -, (Otxr) -, (Drd2) -, (Avpr1a) -*<br>*PFC: (Ar) -, (Esr1) -, (Oprm1) -, (Otxr) -, (Drd2) -, (Avpr1a) -*<br>*NAc: (Ar) -, (Esr1) -, (Oprm1) -, (Otxr) -, (Drd2) -, (Avpr1a) -*<br>*LS: (Ar) -, (Esr1) -, (Oprm1) -, (Otxr) -, (Drd2) -, (Avpr1a) -*	[7]
Fox River Mixture		Lever press latency ↑<br>Errors to Criterion -<br>Reversal Error responses ↓<br>Perseverative Errors -<br>Regressive Errors -<br>Never Reinforced Errors -<br>Reversal Perseveration Error responses ↓	Lever press latency -<br>Errors to Criterion -<br>Reversal Error responses -<br>Perseverative Errors -<br>Regressive Errors -<br>Never Reinforced Errors -<br>Reversal Perseveration Error responses -	[8]
Aroclor 1248		Exploration/Activity ↑	Exploration/Activity -	[9]

(-) No change; (↑) Significant Increase; (↓) Significant Decrease
PFC, Prefrontal Cortex; NAc, Nucleus Accumbens; MPOA, Medial Preoptic Area; LS, Lateral Septum; Ar, Androgen Receptor; Esr1, estrogen receptor alpha; Oprm1, mu opioid receptor; Oxtr, oxytocin receptor; Drd2, dopamine receptor D2; Avpr1a, vasopressin receptor 1a; E_2, Estradiol; P_4, Progesterone; T, Testosterone; T3, Triiodothyronine; T4, Thyroxine

References

1. Peters, M. D.; Godfrey, C. M.; Khalil, H.; McInerney, P.; Parker, D.; Soares, C. B., Guidance for conducting systematic scoping reviews. *Int J Evid Based Healthc* **2015,** *13* (3), 141-6.
2. Rethlefsen, M. L.; Farrell, A. M.; Osterhaus Trzasko, L. C.; Brigham, T. J., Librarian co-authors correlated with higher quality reported search strategies in general internal medicine systematic reviews. *J Clin Epidemiol* **2015,** *68* (6), 617-26.
3. Page, M. J.; McKenzie, J. E.; Bossuyt, P. M.; Boutron, I.; Hoffmann, T. C.; Mulrow, C. D.; Shamseer, L.; Tetzlaff, J. M.; Akl, E. A.; Brennan, S. E.; Chou, R.; Glanville, J.; Grimshaw, J. M.; Hrobjartsson, A.; Lalu, M. M.; Li, T.; Loder, E. W.; Mayo-Wilson, E.; McDonald, S.; McGuinness, L. A.; Stewart, L. A.; Thomas, J.; Tricco, A. C.; Welch, V. A.; Whiting, P.; Moher, D., The PRISMA 2020 statement: An updated guideline for reporting systematic reviews. *BMJ* **2021,** *372*, n71.
4. Schneider, K.; Schwarz, M.; Burkholder, I.; Kopp-Schneider, A.; Edler, L.; Kinsner-Ovaskainen, A.; Hartung, T.; Hoffmann, S., "ToxRtool", a new tool to assess the reliability of toxicological data. *Toxicol Lett* **2009,** *189* (2), 138-44.
5. European Commission, Toxrtool - toxicological data reliability assessment tool. https://ec.europa.eu/jrc/en/scientific-tool/toxrtool-toxicological-data-reliability-assessment-tool. 2019, Accessed: August 13, 2021.
6. Bell, M. R.; Thompson, L. M.; Rodriguez, K.; Gore, A. C., Two-hit exposure to polychlorinated biphenyls at gestational and juvenile life stages: 1. Sexually dimorphic effects on social and anxiety-like behaviors. *Horm Behav* **2016,** *78*, 168-77.
7. Bell, M. R.; Hart, B. G.; Gore, A. C., Two-hit exposure to polychlorinated biphenyls at gestational and juvenile life stages: 2. Sex-specific neuromolecular effects in the brain. *Mol Cell Endocrinol* **2016,** *420*, 125-37.
8. Monaikul, S.; Eubig, P.; Floresco, S.; Schantz, S., Strategy set-shifting and response inhibition in adult rats exposed to an environmental polychlorinated biphenyl mixture during adolescence. *Neurotoxicol Teratol* **2017,** *63*, 14-23.
9. Lombardo, J. P.; Berger, D. F.; Hunt, A.; Carpenter, D. O., Inhalation of polychlorinated biphenyls (PCB) produces hyperactivity in rats. *J Toxicol Environ Health A* **2015,** *78* (18), 1142-53.
